# Cesarean effects on adolescents’ birth experiences: counterfactual analysis

**DOI:** 10.1590/2237-6089-2019-0102

**Published:** 2020-08-14

**Authors:** John P. Connolly, Cheryl Anderson

**Affiliations:** 1 University of Texas at Arlington ArlingtonTX United States of America University of Texas at Arlington , Arlington , TX , United States of America .

**Keywords:** Counterfactual causal effects, moderated mediation, birth experience, adolescent mothers, birth trauma, depression

## Abstract

**Introduction:**

The birth experience of adolescents is understudied even though they are a particularly vulnerable population to experience a negative birth event, given that they exhibit many known risk factors.

**Objective:**

To ascertain whether a cesarean birth mediates the impact of infant complications on the birth experience of adolescent mothers.

**Methods:**

Using a secondary analysis of data collected from 303 postpartum adolescents previously evaluated for depression and post-traumatic stress, we employed counterfactual causal analysis to determine if delivery type mediated the birth experience at different levels of depression. Noted limitations pertain to methodological assumptions and computational feasibility as well as potential sample bias.

**Results:**

We found that the mediating effect of delivery mode depended on the adolescent’s depression level as well as on the specific operationalization of the birth experience. At low levels of depression, the odds of a negative birth appraisal were reduced by around 30% when operationalized as a single item subjective rating. In contrast, at high levels of depression, the odds of a negative birth experience increased by 80% when operationalized as an Impact of Event Scale (IES) subconstruct.

**Conclusion:**

Depression level plays a pivotal role in moderating how delivery mode mediates the birth experience. The direction of impact also depends on how the birth experience is operationalized.

## Introduction

A woman’s birth experience is multidimensional and complex, defined by an interplay of tangible events and resulting perceptions. One way of assessing the birth experience is via a conscious perceptual rating. Other methods of assessment are based on responses addressing different aspects of the experience, including detection of subjective distress and trauma impact. Adolescent mothers are a particularly vulnerable population to report a negative birth event, given an increased likelihood to experience several recognized risk factors observed among adult samples. Specific risks include depression, prior trauma, a lack of information/awareness of events taking place during labor and birth, feelings of loss of control and powerlessness, limited support, unanticipated pain levels, and infant complications. ^1 - 6^

Type of birth has also received attention as a factor that influences a woman’s perception of birth and distress level. ^1 , 2^ More than 32% of women in the United States experience a cesarean birth (CB) annually, and rates continue to rise worldwide. ^7^ A common reason for a primary CB is fetal distress, but several other reasons exist. ^8^ Experiencing either a planned or unplanned CB can evoke negative birth feelings of varying distress levels. ^1 , 9 , 10^ Systematic reviews have noted the influence of type of delivery upon the development of post-traumatic stress symptoms and post-traumatic stress disorder (PTSD). ^11 - 13^ Yet, contrary studies either offer no evidence supporting operative birth as an important predictor of a negative birth experience, ^6 , 14 - 16^ or show preference for a CB. ^17^

Despite the inconsistent findings available in the literature, a link has been identified between CB distress and birth perception, and a negative perception of birth has been recognized as an independent risk factor for the development of PTSD. ^12 , 18 , 19^ Symptoms reflective of PTSD are associated with poor mother-infant bonding, future infertility, an increased fear of childbirth and voluntary CBs in subsequent pregnancies. ^1 , 20 , 21^

Adolescents are recognized to be more prone to experience prenatal and postpartum depression, infant complications, and prior traumas than adults. ^22 - 27^ Demographic data such as age (younger) and race/ethnicity may also indirectly impact one’s birth experience. ^19^ Specifically, minority adolescents may be more prone to report a negative birth experience because of an increased prevalence of several risk factors, ^28^ and notably Black childbearing adult women have been shown to be at exceptional risk for a CB. ^29^ In the Unites States, while comparable CB rates for adolescents and adults are reported at around one in three, adolescents globally are at greater risk for a CB. ^29 - 31^ Yet, despite apparent risks and the high rate of CBs globally, little has been written on the birth experience of adolescent mothers, and the mediating effect of delivery type on the birth event of adolescent mothers with infant complications is unexplored.

In this paper, we focus on a primarily minority group of adolescent mothers with infant complications and, as such, at risk for a negative birth experience. In such circumstances, infant complications may have led to an unplanned CB, which in turn may have further impacted the overall birth experience. The question we address is: Does having a CB mitigate the effect of infant complications on the adolescent’s birth experience? This question positions delivery type as a possible mediating variable through which infant complications influence the experience. We assess this possibility using counterfactual causal analysis and further leverage the potential outcome framework to ascertain whether any such effect is sensitive across levels of depression, which has been recognized to impact the experience for childbearing women. ^11 , 12^

## Methods

This secondary analysis used available data previously collected for an institutional review board-approved longitudinal study exploring the mental health of postpartum adolescents. In the parent study, initial data related to the birth experience were collected via surveys at 72 hours postpartum from 303 Spanish- and English-speaking adolescents aged 13-19 years old. Inclusion/exclusion criteria were related to language and age only. Two measures of birth experience were considered, one pertaining to the mother’s overall birth perception, and another relating to the subjective distress (or trauma impact) of birth (see operationalization of outcome variables below for additional discussion of tools and Table 1 for sample characteristics). Surveys were considered appropriate to the adolescents’ mean age and educational level; however, researchers remained present in the room with the teens in case questions arose. Complete data from 273 adolescents formed the sample for the current study. Additional details for the parent study are discussed elsewhere. ^32^

**Table 1:** Sample descriptive statistics (n = 273)

Variable	n	%
Type of birth: cesarean	63	23
Infant complications	60	21.9
Unplanned pregnancy	187	65.4
Parity (first living infant)	213	78
Prior trauma	58	21.2
Marital status (single)	257	84.8
		
Race/ethnicity		
White	31	10.6
Black	75	25.6
Hispanic	183	62.5
		
Partner presence	160	58.6
Education level, mean and SD	11.32	1.52
Age, mean and SD	17.86	1.38
Depression, mean and SD	6.04	4.80
Minor/major depression	82	30
		
Prenatal rating (“usually happy”)	162	60
		
Avoidance (≥ 20)	44	16
		
Birth appraisal, mean and SD	5.10	2.90
≥ 7 (negative)	99	37

SD = standard deviation

It is known that the “typical” birth experience for women with birth complications is at risk of being more negative than the birth experience of women without infant complications. ^19^ It is also known that the “typical” delivery mode for women with infant complications may be a CB. ^8^ The question we set out to address is whether the typical delivery mode mediates the impact of infant complications on the birth experience, perhaps mitigating the expected negative outcome. To come closer to the counterfactual technical formulation that follows, our goal was to contrast two different expected outcomes: the first is the birth experience of adolescents with infant complications who had a “typical” delivery method (CB); the second is the birth experience of adolescents with infant complications under the counterfactual scenario that their delivery method had been – instead – a delivery method “typical” of adolescents “with no infant complications” (vaginal birth). If the expected outcome under the latter condition is more negative than the former, then this would suggest that delivery mode – in this context, a CB – has some mitigating effect on the impact of infant complications; in short, that the experience would have been (even) worse without the CB. (In support of this point, most women prefer vaginal births, but not all. If a woman gives birth vaginally when expecting or preferring a CB, increased PTSD may result. ^33^ ) Last, given the known contribution of depression upon the birth experience ^11 , 12^ and noted ethnic/racial disparities related to our variables of interest, ^28 - 31^ these two variables were identified as relevant to the analysis as potential confounders. Further, we treated depression as a moderator variable, so that the overall problem we address is one of “moderated mediation,” i.e., one in which the mediated effects vary with levels of depression. (We provide technical definitions of these terms below.) Our previous analyses with this dataset informed our decision to exclude other potential confounders that did not show correlative relationships with the main variables in the current study.

### Counterfactual causal analysis

Our research question called for an analysis of “moderated mediation,” i.e., we hypothesized that there is a mediating impact via mode of delivery type on the birth experience of adolescents with infant complications, but that this mediating effect may in turn depend on the adolescents’ level of depression. A moderator variable is one “that affects the direction and/or strength of the relation between an independent or predictor variable and a dependent or criterion variable,” while a mediator variable is one which “accounts for the relation between the predictor and the criterion.” Moderated mediation thus refers to a situation in which the “mediational effects... vary across the levels” of the moderator. ^34^

While the idea of moderated mediation is not new, there have been various criticisms of the claim that true causal effects are being identified within the traditional structural equation modeling (SEM) approach. ^35 , 36^ Indeed, various complications and ambiguities can arise if one focuses only on the reported indirect effects using traditional SEM. These issues and others have been discussed extensively, pointing to limitations of the traditional approach. ^37^ By now, there is a well-developed literature that integrates causal inference and counterfactual interpretation into mediation analysis. ^38 - 40^ In turn, the flexibility of the causal inference approach has yielded new effects to be defined that are not part of the traditional SEM approach. For our specific purposes, the main benefit of the counterfactual approach is in providing us with a means of incorporating a binary mediator (mode of delivery) into the analysis.

The counterfactual causal analysis framework essentially breaks down the analysis of theorized effects into a missing data problem. In turn, given that certain basic assumptions are valid concerning the data collection process, the solution to estimating causal impacts involves inferring plausible characteristics of the missing (counterfactual) data. Critical to this approach is the notion of an expected outcome (denoted *E* [ ]) of the key variable of interest.

### Variables and expectations of interest

We adopted the following nomenclature in the analysis:

-  
*Y* is the observed outcome, operationalized both as birth appraisal and subjective distress.
-  
*X* is the observed treatment, an indicator of whether the adolescent had infant complications or not (e.g., preterm birth, low birth weight).
-  
*M* is the observed mediator, an indicator of delivery type; in particular, whether the adolescent had a CB or not. (We stress that it is the binary nature of this particular mediator variable that requires a new kind of mediation analysis).
-  
*Z* is the observed moderator, here a continuous measure for level of depression. For this, we used the Edinburgh Postnatal Depression Scale (EPDS) to screen for symptoms of depression, as well as an alternative prenatal depression rating measure which also suggests a depression baseline. The 10-item EPDS has shown adequate reliability results in multicultural and multiethnic adolescent populations. ^41 - 43^ The alternative prenatal score is derived from a single item measure that does not have the same established psychometric properties as EPDS but has greater causal validity in the context of our statistical model, which considers depression as moderating the causal process.
-  
*C* is a set of observed covariates. In particular, the covariates here are confounders to increase the plausibility of certain assumptions required for causal analysis. Two confounders are of note: 1) ethnic/racial background and 2) a history of prior trauma. Published reports suggest that women of diverse ethnic/racial backgrounds are at increased risk for depression symptoms, prior trauma, infant complications, and CB, ^22 , 28 , 29 , 44^ all of which have been associated with a negative birth experience. ^12^ We note that both these confounders come temporally before the birth experience, which adds to the plausibility of their causal role.
-  
*E* [ *Y* (1, *M* (1))] is the expected value of the birth experience for an adolescent with infant complications and whose mode of delivery type is typical of a mother with infant complications.
-  
*E* [ *Y* (1, *M* (0))] is the expected value of the birth experience for an adolescent with infant complications whose mode of delivery type is typical of a mother without infant complications.

A full description of all variables used in the analysis can be found in the online-only supplementary material.

### Applying the counterfactual approach

This analysis centers on the “counterfactual,” or “potential outcome,” *Y*_*i*_ ( *x* ), which denotes the potential outcome that would have been observed for subject *i* had the treatment variable *X* been set to the value *x* , where a value of 0 or 1 denotes respectively the control and treatment groups. In our case, the “treatment” group refers to adolescent mothers with infant complications. The counterfactual concept inherent here is due to the fact that only one of *x* = 1 or 0 will be observed for any one individual; i.e., we only observe an adolescent in one of the two states (infant complications or no infant complications). Since we wish to consider the same individual at both values, we focused on average effects (denoted by the *E* [ ] operator). Thus, while the effect of treatment expressed as *Y*_*i*_ (1) – *Y*_*i*_ (0) cannot be identified, the average effect *E* [ *Y* (1) – *Y* (0)] can.

An example dataset is provided in Table 2 , which presents the potential outcomes for outcome *Y* (birth experience) and mediator *M* (delivery type), but only showing the actual values observed for each adolescent (indexed by *i* ). As such, the first adolescent listed was observed in the treatment group (had infant complications) and was also observed to have a CB ( *M* = 1) with a birth appraisal rating of 10, while the third adolescent was not in the treatment group (i.e., did not have infant complications) but, similarly to the first adolescent, also had a CB – in her case, the birth appraisal rating was 4. Since the counterfactual approach to mediation analysis is central to our purpose of modeling a binary mediator, we provide a brief review of its theoretical underpinnings.

**Table 2:** Potential outcome example

i	X	M(X = 1)	M(X = 0)	Y(X = 1, M = 1)	Y(X = 0, M = 1)	Y(X = 1, M = 0)	Y(X = 0, M = 0)
1	1	1		10			
2	1	0				8	
3	0		1		4		
4	0		0				6
5	1	0				7	
6	0		0				5
7	1	1		8			
8	1	1			2		
							
Average:		0.6	0.3	9.0	3.0	7.5	5.5

### Mediation model

The underlying mediation model which is the foundation for estimating the effects of interest is defined by Equation 1 and Equation 2.

Equation 1:

$$Y_{i}\;=\;\beta_{0}\;+\;\beta_{1}M_{i}\;+\;\beta_{2}X_{i}\;+\;\beta_{3}Z_{i}\;+\;\beta_{4}M_{i}Z_{i}\;+\;\beta_{5}C_{i}\;+\;\varepsilon_{yi}$$

Equation 2:

$$M_{i\;}=\;\gamma {\;}_{0}\;+\;\gamma {\;}_{1}X_{i}\;+\;\gamma {\;}_{2}C_{i}\;+\;\varepsilon_{mi}$$

As such, the expectation of *Y* depends on how the mediator *M* varies under the specified conditions, which implies an integration over *M* , as shown in Equation 3.

Equation 3:

$$E[Y(x,\;M(x\prime )\;|\;C\;=\;c,\;Z\;=\;z]\;=\int_{-\infty }^{+\infty }E[Y|C=c,X=x,M-m,Z=z]\times f(M|C=c,Z=z,X=x')\partial M=\beta_{0}+\beta_{2}x+\beta_{3}z+\beta_{5}c+\left(\beta_{1}+\beta_{4}z\right)\int_{-\infty }^{+\infty }mf\left(M;\;\Upsilon_{0}+\Upsilon_{1}x'+\Upsilon_{2}c,\sigma_{m}^{2}\right)\partial M$$

While there are various direct and indirect effects capable of being defined under the potential outcome framework, our specific interest here was in the conditional total natural indirect effect (TNIE), shown in Equation 4. ^37 , 45^

Equation 4:

$$TNIE=E[Y(1,\;M(1))-Y(1,M(0))\;|\;C\;=\;c,\;Z\;=\;z]\;=\int_{-\infty }^{+\infty }E[Y|C=c,X=1,M=1,Z=z]\times f(M|C=c,Z=z,X=1)\partial M-\int_{-\infty }^{+\infty }E[Y|C=c,X=1,M=0,Z=z]\times f(M|C=c,Z=z,X=0)\partial M$$

In the context of our study, this translates to the difference between those who had infant complications ( *X* = 1) in the expected value of birth appraisal ( *Y* ), based on the adolescents’ typical mode of delivery type under these circumstances ( *M* (1)) compared to the expected value of birth appraisal based on the typical mode of delivery type had there been no infant complications ( *M* (0)), conditional over a range of moderator, *Z* (depression), values.

### Accommodating a binary mediator

The case in which the mediator is itself binary, as is the case here, has also been extensively discussed in the mediation literature. ^46 - 48^ In this case, the process of integration described above is simplified with the integral being replaced with a sum over the two values of *M* and the density *f* being replaced with the probabilities of *M* taking on the values 0 or 1. Ignoring now for simplicity of expression the conditioning on covariates *C* and moderator *Z* , the TNIE can be expressed in terms of a binary mediator as follows (Equation 5).

Equation 5:

$$TNIE\;=\;E[Y(1,\;M(1))\;–\;Y(1,\;M(0))]\;=\;E[Y(1,\;0)]\;(1\;–\;F_{M}(1))\;+\;E[Y(1,\;1)](F_{M}(1))\;–\;[E[Y(1,\;0)](1\;–\;F_{M}(0))\;+\;E[Y(1,\;1)](F_{M}(0))]\;=\;[E[Y(1,\;1)]\;–\;E[Y(1,\;0)]][F_{M}(1)\;–\;F_{M}(0)]$$

where *F*_*M*_ ( *x* ) denotes *P* ( *M* = 1 | *X* = *x* ), with *F* representing either the standard normal or logistic distribution function corresponding to using probit or logistic regression, respectively. ^45^

### Accommodating a binary outcome

If the outcome is also binary, then *E* [ *Y* ] = *P* ( *Y* = 1)]. Letting *F*_*Y*_ ( *x, m* ) denote *P* ( *Y* = 1 | *X* = x, *M* = *m* ), the version of the expression above for a binary outcome is as follows (Equation 6).

Equation 6:

$$TNIE\;=\;E[Y(1,\;M(1))\;–\;Y(1,\;M(0))]\;=\;[F_{Y}(1,\;1)\;–\;F_{Y}(1,\;0)][F_{M}(1)\;–\;F_{M}(0)]$$

The TNIE can also be expressed in terms of an odds ratio (Equation 7).

Equation 7:

$$TNIE\;=\;\frac{P\left(Y_{x_{1}Mx_{1}}=1\right)/\left(1-P\left(Y_{x_{1}M_{x_{1}}}=1\right)\right)}{P\left(Y_{x_{1}Mx_{0}}=1\right)/\left(1-P\left(Y_{x_{1}M_{x_{0}}}=1\right)\right)}$$

The estimated indirect effect and direct effects for a binary outcome usually have non-normal sampling distributions so that a non-symmetric confidence is needed. ^34^ As such, we obtained bootstrap confidence intervals using 1,000 bootstrap draws with maximum-likelihood estimation in the analysis to follow.

## Operationalization of the outcome variable

Here we consider two different aspects of the birth experience measured as birth perception and subjective distress. The first is a straightforward single indicator variable (0 to 10) of the adolescent’s overall appraisal (rating) of the experience from 1 (great) to 10 (awful). Appraisal of the birth experience has been assessed using a one-item measure in several previous studies, with a suggested cutoff of > 6 as indicative of a traumatic birth appraisal. ^6 , 49^ The second aspect of birth is based on the Impact of Event Scale (IES). ^50^ The 15-item IES was developed to determine subjective distress/trauma impact, with numerous researchers emphasizing the IES’s two-factor structure (intrusion and avoidance) as stable over different types of events. ^51^ With this recognized utility as a research tool, the IES was one of the first instruments used among childbearing populations to measure PTSD following birth. ^52 , 53^

Since the IES is a multi-item scale, we assessed its construct validity and found that the 8-item avoidance subscale exhibited stable statistical properties (root mean square error of approximation [RMSEA = 0.06], comparative fit index [CFI = 0.99]); however, neither the 7-item intrusion subscale (RMSEA = 0.11, CFI=0.97), nor the overall 15-item construct did so (RMSEA = 0.13, CFI = 0.84). As such, we used only the avoidance scale from the IES instrument, juxtaposed as an alternative outcome measure to the single-item birth appraisal rating. By definition, avoidance refers to numb feelings relating to birth, or denial of feelings about the birth experience with efforts not to think or talk about the birth. ^54^

## Results

The results for the two estimated models are shown in Tables 3 and 4, respectively. Given that our main interest was in true causal effects that may further vary over a range of moderator values, we emphasize that the two tables do not convey this key information and that our inferences are instead to be drawn from the reported TNIE effects. That being said, the directional effects of the predictors were at least consistent with findings from other studies. In particular, infant complications were predictive of a negative birth appraisal ( Table 3 ), and also were more likely to lead to a CB. Also, Black adolescents were more likely to indicate a negative birth appraisal. By contrast, the results reported in Table 4 show that infant complications did not exhibit a direct effect on avoidance, although an adolescent’s level of depression did have such a direct effect.

**Table 3:** Logistic regression estimates with outcomes for birth appraisal and delivery type

	Estimate	SE	Estimate/SE	p-value
Birth appraisal				
Delivery type	-1.623	0.833	-1.949	0.051
Infant complications	0.903	0.382	2.364	0.018
Depression	0.035	0.034	1.036	0.300
Depression * delivery type	0.097	0.091	1.060	0.289
Race	1.225	0.333	3.679	0.000
Trauma	-0.611	0.395	-1.546	0.122
				
Delivery type				
Infant complications	1.387	0.349	3.977	0.000
Race	0.584	0.366	1.598	0.110
Trauma	-0.359	0.439	-0.817	0.414
				
Intercepts				
Birth appraisal	0.962	0.270	3.564	0.000
Delivery type	1.760	0.243	7.245	0.000

SE = standard error.

**Table 4:** Logistic regression estimates with outcomes for avoidance and delivery type

	Estimate	SE	Estimate/SE	p-value
Avoidance				
Delivery type	-0.248	1.297	-0.191	0.848
Infant complications	-0.556	0.533	-1.043	0.297
Depression	0.105	0.041	2.534	0.011
Depression * delivery type	0.138	0.124	1.105	0.269
Race	0.303	0.409	0.740	0.459
Trauma	0.226	0.444	0.508	0.612
				
Delivery type				
Infant complications	1.387	0.349	3.977	0.000
Race	0.584	0.366	1.598	0.110
Trauma	-0.359	0.439	-0.871	0.414
				
Intercepts				
Avoidance	2.654	0.435	6.104	0.000
Delivery type	1.760	0.243	7.245	0.000

SE = standard error.

**Figure 1:**
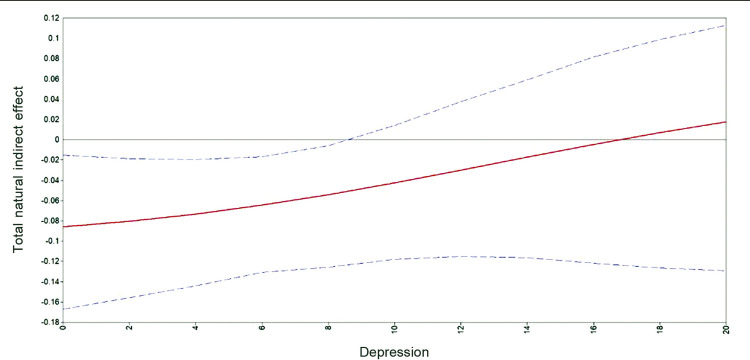
Total natural indirect effect of delivery type on birth appraisal across levels of depression for adolescent mothers with infant complications.

**Figure 2:**
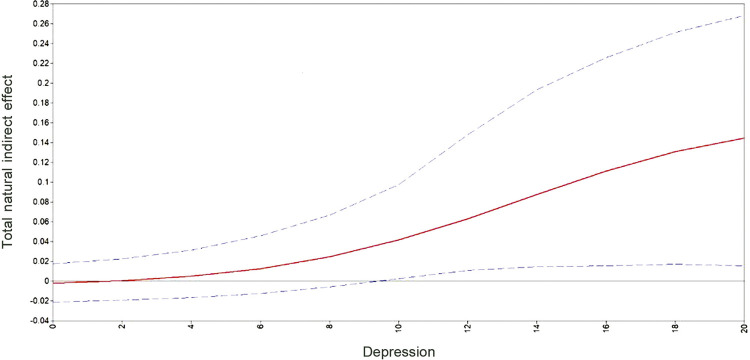
Total natural indirect effect of delivery type on avoidance across levels of depression for adolescent mothers with infant complications.

It is not obvious from these estimates that there was any mediation effect of delivery type on either of the outcome variables, nor whether any such mediation effect varied according to depression level. This requires computation of our main causal effects of interest, specifically the conditional TNIE as defined above, across different levels of depression. These results are depicted in Figures 1 and 2 and show that delivery type does indeed function as a mediator variable but in a manner that depends on 1) the nature of the outcome variable, and 2) the level of depression. Two features of these results are noticeable. First, in the case of birth appraisal, the indirect effect of infant complications via delivery type is only significant at lower levels of depression and is negative. This means that adolescents with infant complications with no, or low levels of, depression tended to report a better birth appraisal if they had a CB. In contrast, the second graph shows an opposite tendency in the case of avoidance (subjective distress), i.e., adolescents with infant complications and high levels of depression tended to report a higher level of avoidance if they had a CB.

The second feature of note is that the effect sizes are low to moderate in either case, which is suggestive of additional research. For example, in the first case, a CB for an adolescent with no depression may reduce the probability of a negative birth appraisal by 2-16 percentage points, which translates to an odds ratio (as defined above) of around 0.7. In the second case, a CB for an adolescent with infant complications and with the highest depression score in our sample was more likely to report a higher level of avoidance by 2-26 percentage points, which translates to an odds ratio of around 1.8. These results were replicated using the prenatal rating measure of depression as the moderator. As with the case above, where birth appraisal was the outcome variable, only for the adolescents who indicated they were “usually happy” did a CB mitigate the impact of infant complications on the birth appraisal evaluation. Likewise, in the case of the avoidance outcome measure, adolescents who were more depressed during pregnancy (specifically here, those who were in the range “sometimes happy” to “always sad”) indicated higher avoidance scores after a CB.

### Assumptions and limitations

The main assumptions and limitations here are of two types; the first pertaining to the causal effect methodology and the second pertaining to our specific research design and sample. Assumptions of the counterfactual approach were summarized by VanderWeele & Vansteelandt. ^55^ These mostly pertain to the need to control for variables that simultaneously affect any pair among the treatment, mediator and outcome variables. We sought to address these issues by the inclusion of confounder variables in our analysis. In addition, these confounders, (prior trauma and ethnicity/race) must not be affected by the exposure (infant complications), which in our case is assured by the natural temporal ordering of these variables. We do, however, acknowledge a potential issue concerning endogeneity, given that our preferred measure of depression (EPDS scale) was contemporaneous with the outcome variables. However, as noted earlier, we replicated our results using a prenatal rating measure of depression, albeit this was a measurement tool of less content validity.

Additional limitations reflect the use of secondary analysis. Measurement choices and procedures for data collection were preset within the parent study. However, the choice of tools to assess birth experience in general represents a challenge, as available instruments range widely in purpose, content, and psychometric properties. ^56^ Baseline depression was not established via a strong measure; however, it has been reported that the occurrence of depression in the last trimester of pregnancy and in the immediate postpartum period is similar. ^57^ Further, given our use of available data, all potential confounders could not be examined, including reason for CB. However, our assumption is that all the CBs reported in our sample were for medical reasons and unplanned, given age and knowledge of birth options by the typical adolescent. Last, sample biases may include age and ethnic characteristics among small samples, especially for white adolescents.

## Discussion

The role of CB on the mental state of women has been investigated by various scholars, often finding a negative impact. Available literature, however, notably considers primarily adults and often overlooks confounders such as race/ethncitiy. ^19^ In addition, the methodology employed is limited in terms of identifying true causal effects and the role of CB as a mediator of birth outcomes. Our study is unique in several respects: 1) we examined a population, specifically adolescent mothers, for which CBs are most likely to be experienced due to medical reasons, and not pre-planned (elective); 2) we considered various aspects of the birth experience, recognizing that a plethora of different measuring instruments is currently being used; and 3) we assessed the role of CB using modern counterfactual analysis, which allows one to identify true causal effects.

The non-parametric nature of the counterfactual analysis formulation enables consideration of outcome and mediator variables that are not necessarily normally distributed. This was crucial for our purposes in being able to treat CB as a mediator, as it is inherently a binary variable. With regard to the outcome variable, while we only presented results using binary measures of the birth appraisal and avoidance outcome variables, we also performed the same analysis with the continuous versions of these variables and obtained the same substantive results. Our choice of presenting the results for the binary outcome variables also lent more naturally to expressing effect sizes in terms of probabilities and odds ratios.

Results of the study also provided several clinical insights relevant to the childbearing adolescent which lend support to existing literature but also expand current thought. Infant complications were found to predict both CB and a negative birth experience. However, birth appraisal varied according to level of depression. Adolescents with infant complications but without reported depression who experienced a CB appraised their birth experience better than in a scenario with no CB. This finding may suggest that adolescents without depression are better able to perceive the value/need of a CB to save her own or her infant’s life than the depressed teen. While adolescents, more likely than adults, may rate both vaginal and CBs as positive, ^58^ the role of depression as a contributor to a negative birth experience and potential PTSD is important to acknowledge to guide assessments and treatment. Adolescent depression rates both prenatally and after birth are higher than reported rates among adults and draws attention to the possible increased vulnerability of childbearing adolescents. ^59^

Black race/ethnicity also contributed to a negative birth appraisal. Race/ethnicity and age are frequently overlooked as potential contributors to birth trauma, and when examined, findings are inconsistent. ^19^ Risk factors related to a negative birth experience among primarily adults have been found to often characterize the Black woman, including higher rates of CB and infant complications. ^29 , 31 , 60^ Additional patient-level health factors, quality of physician-provider interaction, and patient preferences are also recognized as linked to infant complications and higher rates of CB among Black women. ^31^ Therefore, a combination of factors may have increased the likelihood for the Black adolescent to experience a negative birth event. Side analyses did indicate a significantly higher percentage of infant complications and CB rates among the Black adolescents; however, additional research is needed to further assess the vulnerability of the Black adolescent to a negative birth experience.

Neither race/ethnicity nor infant complications showed a direct effect on the avoidance outcome measure; yet, depressed adolescents with infant complications and CB had higher avoidance scores than they would have had without a CB, suggesting that, if the adolescent is highly depressed, a CB will tend to accentuate feelings and actions associated with avoidance (such as numb feelings, denial of feelings, and efforts not to think or talk about the birth). While perhaps such actions may be within the nature of depression itself, other factors for these young mothers can also exist. While already facing multiple challenges above and beyond the transition to motherhood, a CB may bring additional distress due to unexpected birth consequences such as an adverse infant condition and admission to neonatal intensive care unit (NICU), as well as increased postpartum pain and longer hospitalization and recovery time following birth. Studies have described maternal mental health consequences to preterm birth and NICU admissions. ^61^ While shock and disbelief due to unexpected infant complications or CB is not an unlikely reaction by any age woman, a younger adolescent may have more difficulties in managing the situation, especially if depressed. A high rate of CB has been recognized and studies are being conducted to aid in its reduction ^7^ ; yet, minimal advance has been seen related to hospital policy and state legislation supporting mandatory education and/or routine assessment of depression for childbearing women. ^62 , 63^ Given the fact that the present findings illustrate an interaction between depression and delivery mode as an influence on both birth appraisal and avoidance, and given a recognized possibility of subsequent mental health concerns from additional works, suggested research and clinical practice actions could be informed by this study as well as by future research adopting the counterfactual approach.

## Conclusion

Using counterfactual causal analysis, we found evidence that delivery type does indeed exhibit a mediating impact on the birth experience of adolescent mothers with infant complications, but that the size and direction of this effect depends in turn on how the birth experience is operationalized as well as on existing levels of depression. Specifically, we found opposite effects when the outcome was measured in terms of a conscious appraisal of the overall birth experience as compared to a subjective distress “avoidance” reaction. For adolescent women with low levels of depression, a CB leads to a better birth appraisal than it would without a CB. Yet for adolescent women with moderate to high levels of depression, a CB leads to a higher avoidance reaction than would without a CB. In summary, both potential positive and negative perceptions emerge from giving birth by CB, depending on depression levels, and essential assessments of high-risk adolescents can guide immediate and future assessments and treatment.
