# Supplementary material for: Cesarean effects on adolescents’ birth experiences: counterfactual analysis
Source: Trends Psychiatry Psychother. 2020 Aug 14;42(4):318–28. doi: 10.1590/2237-6089-2019-0102 (PMC7879090; doi:10.1590/2237-6089-2019-0102)
Supplement: Supplementary file 1 [file 2238-0019-trends-42-04-0318-suppl.pdf]

Online-only supplementary material: Measurements

Independent variable:
Infant complications: coded 1 if the adolescent's number of gestational weeks was under 37, or if the birth weight was under 2,500 grams, or if some other complication with the newborn was reported at birth. As such, 60 (21%) adolescents were coded as 1.

Mediator variable:
Delivery mode: coded 0 for vaginal or 1 for CB. Sixty-three (22%) adolescents gave birth by cesarean.

Moderator variable:
Depression: coded on a scale of 0 to 30 based on the Edinburgh Postnatal Depression Score (EPDS). However, the range of scores in our dataset was only from 0 to 20. The mean score was 6.04, and the standard deviation 4.8. Four possible responses for each question are scored 0-3, creating a range between 0 and 30. A score of 10 or more indicates symptoms suggestive of minor depression; a score of 13 or more indicates symptoms of major depression.[1] We also used an alternative prenatal depression measure. This was a single-item measure based on the question "How do you remember feeling during pregnancy?" It was coded 1-5, where 1 indicated "usually happy" and 5 "always sad."

Confounder variables:
Prior trauma: coded 1 if the mother reported either a history of child abuse, was currently or has been abused by a partner, or has experienced other previous traumatic events; otherwise coded 0. As such, 58 adolescents (19%) were coded as having experienced prior trauma.
Ethnicity: coded 1 if the mother was African American, and 0 otherwise. As such, 75 (25%) of the adolescents were coded as 1.

Outcome variables:
Birth appraisal: rating of the childbirth experience ranging from 1 to 10, where 10 represented "awful or extremely traumatic." The mean score was 5.1 and the standard deviation 2.9. Nearly two-thirds of the 188 adolescents (65%) indicated a positive (not traumatic) birth appraisal, while 103 (35%) of the adolescents indicated a negative (traumatic) birth appraisal.
Avoidance: based on items 2, 3, 7, 8, 9, 12, 13, and 15 of the 15-item Impact of Event Scale listed (IES), below[2]:

1. **ies1:** coded 0 if answered "not at all," 1 if answered "rarely," 3 if answered "sometimes," and 5 if answered "often" in response to the statement "<u>I thought about it when I didn't mean to</u>."
2. **ies2:** coded 0 if answered "not at all," 1 if answered "rarely," 3 if answered "sometimes," and 5 if answered "often" in response to the statement "<u>I avoided letting myself get upset when I thought about it or was reminded about it</u>."
3. **ies3:** coded 0 if answered "not at all," 1 if answered "rarely," 3 if answered "sometimes," and 5 if answered "often" in response to the statement "<u>I tried to remove it from memory</u>."

4. **ies4:** coded 0 if answered "not at all," 1 if answered "rarely," 3 if answered "sometimes," and 5 if answered "often" in response to the statement "I had trouble falling asleep or staying asleep because of pictures or thoughts about it that came to my mind."
5. **ies5:** coded 0 if answered "not at all," 1 if answered "rarely," 3 if answered "sometimes," and 5 if answered "often" in response to the statement "I had waves of strong feelings about it."
6. **ies6:** coded 0 if answered "not at all," 1 if answered "rarely," 3 if answered "sometimes," and 5 if answered "often" in response to the statement "I had dreams about it."
7. **ies7:** coded 0 if answered "not at all," 1 if answered "rarely," 3 if answered "sometimes," and 5 if answered "often" in response to the statement "I stayed away from reminders about it."
8. **ies8:** coded 0 if answered "not at all," 1 if answered "rarely," 3 if answered "sometimes," and 5 if answered "often" in response to the statement "I felt as if it hadn't happened or was unreal."
9. **ies9:** coded 0 if answered "not at all," 1 if answered "rarely," 3 if answered "sometimes," and 5 if answered "often" in response to the statement "I tried not to talk about it."
10. **ies10:** coded 0 if answered "not at all," 1 if answered "rarely," 3 if answered "sometimes," and 5 if answered "often" in response to the statement "Pictures about it popped into my mind."
11. **ies11:** coded 0 if answered "not at all," 1 if answered "rarely," 3 if answered "sometimes," and 5 if answered "often" in response to the statement "Other things kept making me think about it."
12. **ies12:** coded 0 if answered "not at all," 1 if answered "rarely," 3 if answered "sometimes," and 5 if answered "often" in response to the statement "I was aware that I still had a lot of feelings about it, but I didn't deal with them."
13. **ies13:** coded 0 if answered "not at all," 1 if answered "rarely," 3 if answered "sometimes," and 5 if answered "often" in response to the statement "I tried not to think about it."
14. **ies14:** coded 0 if answered "not at all," 1 if answered "rarely," 3 if answered "sometimes," and 5 if answered "often" in response to the statement "Any reminder brought back feelings about it."
15. **ies15:** coded 0 if answered "not at all," 1 if answered "rarely," 3 if answered "sometimes," and 5 if answered "often" in response to the statement "My feelings about it were kind of numb."

References

1. Cox JL, Holden JM, Sagovsky R. Detection of postnatal depression: development of the 10-item Edinburgh Postnatal Depression Scale. Br J Psychiatry. 1987;150:782-6.
2. Horowitz M, Wilner N, Alvarez A. Impact of Event Scale: a measure of subjective distress. Psychosom Med. 1979;41:209-18.
